# Performance of a deep-learning-based lung nodule detection system using 0.25-mm thick ultra-high-resolution CT images

**DOI:** 10.1007/s11604-025-01828-z

**Published:** 2025-07-07

**Authors:** Haruka Higashibori, Wataru Fukumoto, Sayaka Kusuda, Kazushi Yokomachi, Hidenori Mitani, Yuko Nakamura, Kazuo Awai

**Affiliations:** 1 Department of Diagnostic Radiology, Graduate School of Biomedical and Health Science, Hiroshima University, 1-2-3 Kasumi, Minamiku, Hiroshima 734-8551 Japan; 2 Department of Diagnostic Radiology, Hiroshima University Hospital, 1-2-3 Kasumi, Minamiku, Hiroshima 734-8551 Japan

**Keywords:** Artificial intelligence, Computer-aided detection, Ultra-high-resolution CT, Lung nodule

## Abstract

**Purpose:**

Artificial intelligence (AI) algorithms for lung nodule detection assist radiologists. As their performance using ultra-high-resolution CT (U-HRCT) images has not been evaluated, we investigated the usefulness of 0.25-mm slices at U-HRCT using the commercially available deep-learning-based lung nodule detection (DL-LND) system.

**Materials and methods:**

We enrolled 63 patients who underwent U-HRCT for lung cancer and suspected lung cancer. Two board-certified radiologists identified nodules more than 4 mm in diameter on 1-mm HRCT slices and set the reference standard consensually. They recorded all lesions detected on 5-, 1-, and 0.25-mm slices by the DL-LND system. Unidentified nodules were included in the reference standard. To examine the performance of the DL-LND system, the sensitivity, and positive predictive value (PPV) and the number of false positive (FP) nodules were recorded.

**Results:**

The mean number of lesions detected on 5-, 1-, and 0.25-mm slices was 5.1, 7.8 and 7.2 per CT scan. On 5-mm slices the sensitivity and PPV were 79.8% and 46.4%; on 1-mm slices they were 91.5% and 34.8%, and on 0.25-mm slices they were 86.7% and 36.1%. The sensitivity was significantly higher on 1- than 5-mm slices (*p* < 0.01) while the PPV was significantly lower on 1- than 5-mm slices (*p* < 0.01). A slice thickness of 0.25 mm failed to improve its performance. The mean number of FP nodules on 5-, 1-, and 0.25-mm slices was 2.8, 5.2, and 4.7 per CT scan.

**Conclusion:**

We found that 1 mm was the best slice thickness for U-HRCT images using the commercially available DL-LND system.

## Introduction

Numerous artificial intelligence (AI) algorithms for lung nodules detection, i.e. computer-aided detection (CAD), have been developed to assist radiologists. AI algorithms demonstrated high sensitivity (80–97%) for lung nodule detection using the public database Lung Image Database Consortium and Image Database Resource Initiative (LIDC/IDRI) and deep-learning-based algorithms outperformed lung nodule detection by radiologists, in particular for smaller nodules [1, 2]. However, more clinical data are needed to ascertain their performance as the scan conditions and vendors can affect it [3]. There are few reports on the performance of AI algorithms for lung nodule detection systems using the latest CT technologies such as ultra-high-resolution CT (U-HRCT) and photon-counting detector CT (PCD-CT).

U-HRCT yielded a spatial resolution of 0.14 mm in metal slit-phantom experiments with a minimum focus size of 0.4 × 0.5 mm for the X-ray tube and 0.25 × 0.25 mm for the detector element [4, 5]. Currently, 0.25-mm slices provide the highest spatial resolution among conventional energy-integrating detector CT scanners. This facilitates the visualization of anatomical structures and the detection of abnormal CT findings such as ground-glass opacity, emphysema, and interlobular septal thickening on U-HRCT images [6, 7]. Xu Y. et al. found that U-HRCT identified very small emphysematous lesions in smokers [8]. A study of COVID-19 pneumonia by Iwasawa T. et al. suggested that U-HRCT is useful for the detection of abnormalities accompanied by local lung volume loss, thereby indicating disease severity [9].

Thinner slices increase the number of slices per scan and the reading workload [10]. Besides, image noise also increases with thinner slices. These factors may lead to an increase in the missing nodules and diagnostic errors. Therefore, AI technologies are expected to solve this problem. There is evidence that AI algorithms for lung nodule detection depend highly on the section thickness and the reconstruction interval. Partial volume effects were reduced when thin sections and reconstruction intervals less than 1 mm were applied [11, 12]. However, there are no reports on the performance of AI algorithms when the U-HRCT slice thickness is 0.25 mm.

We examined the performance of the commercially available deep-learning-based lung nodule detection (DL-LND) system using different slice thicknesses for U-HRCT images under the hypothesis that the 0.25-mm slices improve its performance.

## Materials and methods

This retrospective study was approved by our institutional review board; prior informed consent was waived.

### Subjects

Between July and December 2023, we initially enrolled 66 patients who underwent U-HRCT for lung cancer or suspected lung cancer. We then excluded three patients who underwent neoadjuvant chemotherapy due to morphological nodal changes. Consequently, the study population consisted of 63 patients (35 males, 28 females; median age 74 years, range 41–88 years); 58 patients were pathologically diagnosed with lung cancer (*n* = 52), metastatic tumor (*n* = 3), mycobacterial infection (*n* = 1), granulomatosis with polyangiitis (*n* = 1), and organizing pneumonia (*n* = 1) by surgery. One patient was diagnosed with bronchoscopy and underwent chemoradiation therapy. One patient underwent radiation therapy without pathological examination. Although one patient was diagnosed with lung cancer, he was followed up due to severe liver dysfunction. Two other patients were followed up without treatment because inflammation was suspected. There were 12 patients with emphysema and 3 patients with interstitial pneumonia.

### U-HRCT scanning

All subjects were scanned with a 160-detector U-HRCT scanner (Aquilion Precision, Canon Medical Systems) using super-high-resolution mode without contrast material. The parameters for helical scans were tube voltage 120 kV, tube current automatic exposure control, preset noise 25 Hounsfield units (HU), rotation time 0.5 s, beam collimation 0.25 mm × 160 rows, focus size 0.6 × 0.6 mm, pitch factor 0.806, matrix size 512 × 512, field of view (FOV) 500 × 500 mm. The images were reconstructed at a slice thickness/interval of 5/5-, 1/1-, and 0.25/0.25-mm. Hybrid iterative reconstruction (AIDR 3D Standard FC52) was applied. The reconstructed FOV depended on the patient’s body size. For 63 patients, the mean was 365 × 365 mm (SD: 30). The mean computed tomography dose index and the dose length product on the 63 U-HRCT scans were 17.8 mGy (SD: 6.9) and 945.5 mGy・cm (240.9), respectively.

### Reference standards

Two board-certified radiologists identified nodules more than 4 mm in diameter on 1-mm HRCT slices independently and set the reference standard consensually. The 4-mm cutoff size was set based on Lung-RADS and earlier studies [1, 13]. The nodule diameter and subtype were also recorded. For the measurement of nodules less than 10 mm, the average of long-axis and short-axis diameters was adapted, and for those greater than 10 mm, the maximum diameter was adapted based on the recommendations for measuring pulmonary nodule at CT from the Fleischner Society [14]. The nodules were classified as solid-, part-solid-, and ground-glass nodules (GGNs).

The board-certified radiologists reviewed all nodules detected by the DL-LND system and unidentified nodules whose diameter exceeded 4 mm were recorded; nodules that were missed by the radiologists and detected by the DL-LND system were also included in the reference standard.

### Deep-learning-based lung nodule detection

The DL-LND system attached to the SYNAPSE SAI viewer V2.4 (FUJIFILM Medical Co., Ltd) was employed to inspect 5-, 1-, and 0.25-mm-thick slice images. The vendor recommends non-contrast chest CT images of adults (matrix size 512 × 512, slice thickness and interval 5 mm, lung kernel). The DL-LND instrument was programmed to identify solid nodules larger than 3 mm and sub-solid nodules (part-solid nodules and GGNs) larger than 5 mm using a green square.

### Evaluation of the DL-LND system

The total and mean number of lesions detected by the DL-LND system per CT scan on 5-, 1-, and 0.25-mm slices were recorded. Differences in the number of lung nodules detected by the DL-LND system according to the presence or absence of underlying emphysema and interstitial pneumonia were investigated between the same slice thickness. The system’s sensitivity and the PPV, the total- and the mean number of FP nodules per CT scan were evaluated at the three slice thicknesses. Its sensitivity for nodules and the PPV were determined based on their percentage in the reference standards. To examine the utility of thinner slice thickness images, the sensitivity for small nodules (4–10 mm) and sub-solid nodules were also investigated. The processing time of the DL-LND system to detect lung nodules on 5-mm, 1-mm, and 0.25-mm slices was manually measured.

### Statistical analysis

Statistical differences between the number of nodules and FP nodules and the image noise on 5-, 1-, and 0.25-mm slices were determined with the paired *t* test. Student's t test was used to evaluate the differences in the number of lung nodules detected by the DL-LND system according to the presence or absence of underlying emphysema and interstitial pneumonia. The chi-squared test was applied to assess statistical differences in the sensitivity and PPV of the three slice thicknesses. All analyses were with a statistical software package (JMP Pro 18.1.0); *p* values less than 0.05 were considered statistically significant.

## Results

### Reference standards

The radiologists detected 180 nodules on 1-mm slices; 8 were missed by the radiologists but detected by the DL-LND system. Finally, the reference standard contained 188 nodules (median size 7 mm, range 4–66 mm, 88 solid, 41 part-solid nodules, and 59 GGNs). The details of the reference standard nodules are shown in Table 1. There were no true positive nodules other than the reference standard nodules detected only on 5- and 0.25-mm slices.

**Table 1:** Details of the reference standard nodules

	Number	Median size	Range	Size distribution (mm)			
		(mm)	(mm)	4–10	11–20	21–30	31-
Solid nodules	88	8	4–66	49	16	16	7
Part-solid nodules	41	Total size: 10	5–36	21	16	3	1
		Solid portion: 4	1–23				
Ground glass nodules	59	6	4–21	51	6	2	0
Total	188	7	4–66	121	38	21	8

### Evaluation of the DL-LND system

A summary of the results is shown in Table 2.

**Table 2:** A summary of the results

Evaluation of the DL-LND system	Slice thickness		
	5 mm	1 mm	0.25 mm
Mean number of lesions per CT scan	5.1	7.8	7.2
Sensitivity (%)	79.8	91.5	86.7
PPV (%)	46.4	34.8	36.1
Mean number of FP nodules per CT scan	2.8	5.2	4.7

*DL-LND* Deep-learning-based lung nodule detection

*PPV* Positive predictive value

*FP* False positive

**Fig. 1:**
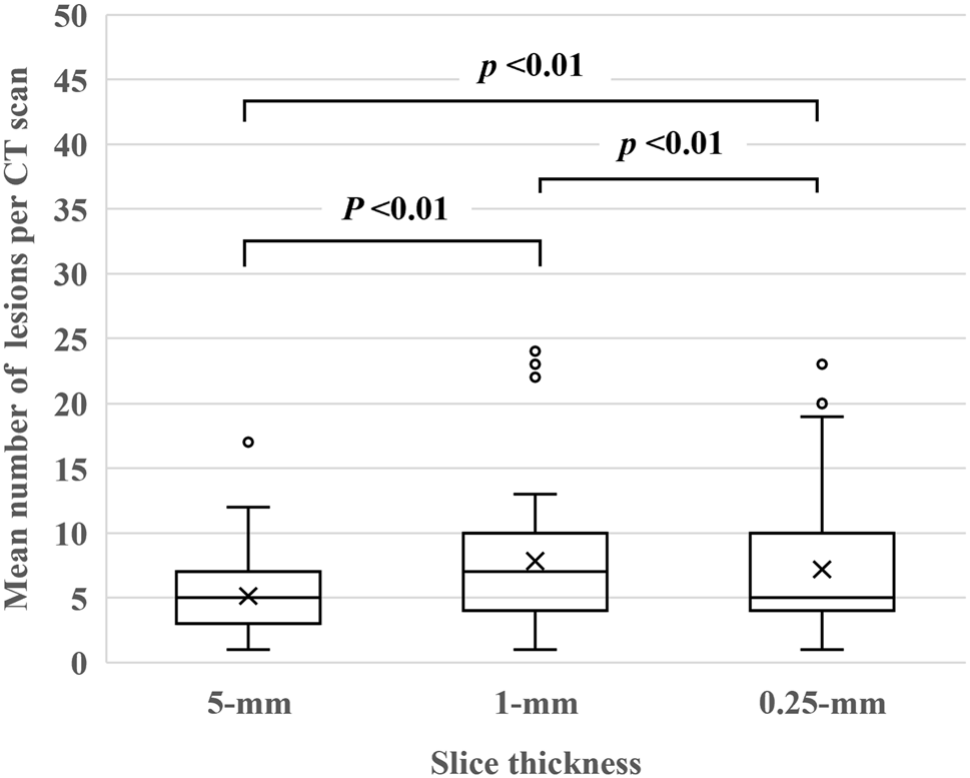
The mean number of lesions on 5-, 1-, and 0.25-mm slices were 5.1 (SD: 2.8), 7.8 (5.5), and 7.2 (4.8) per CT scan; the difference among the three slice thicknesses was significant (all: *p* < 0.01)

The total number of lesions detected by the DL-LND system was 323 on 5-, 494 on 1-, and 452 on 0.25-mm slices. The mean number of lesions on 5-, 1-, and 0.25-mm slices were 5.1 (SD: 2.8), 7.8 (5.5), and 7.2 (4.8) per CT scan; the difference among the three slice thicknesses was significant (all: *p* < 0.01) (Fig. 1). There were no significant differences in the number of lung nodules detected by the DL-LND system according to the presence or absence of underlying emphysema and interstitial pneumonia (5 mm; *p* = 0.75, 1 mm; *p* = 0.74, 0.25 mm; *p* = 0.45).

**Fig. 2:**
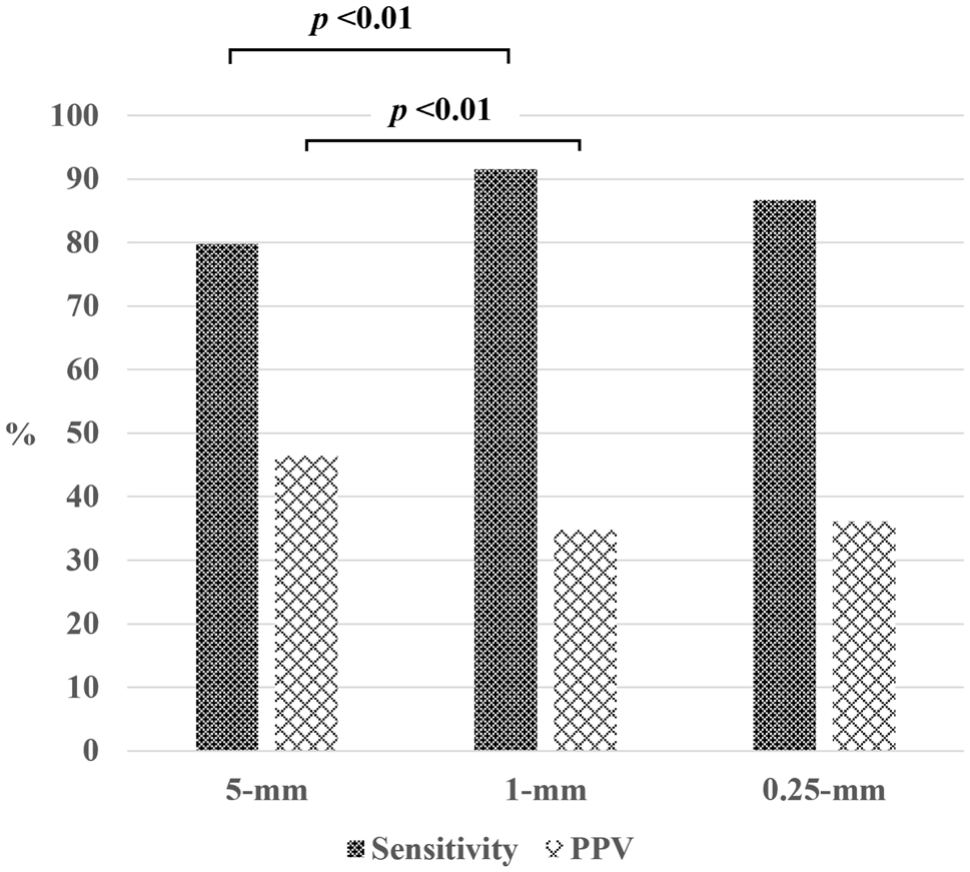
The sensitivity and the PPV on the three slice thicknesses (%). The sensitivity was significantly higher on 1- than 5-mm slices (*p* < 0.01). The PPV was significantly lower on 1- than 5-mm slices (*p* < 0.01)

**Fig. 3:**
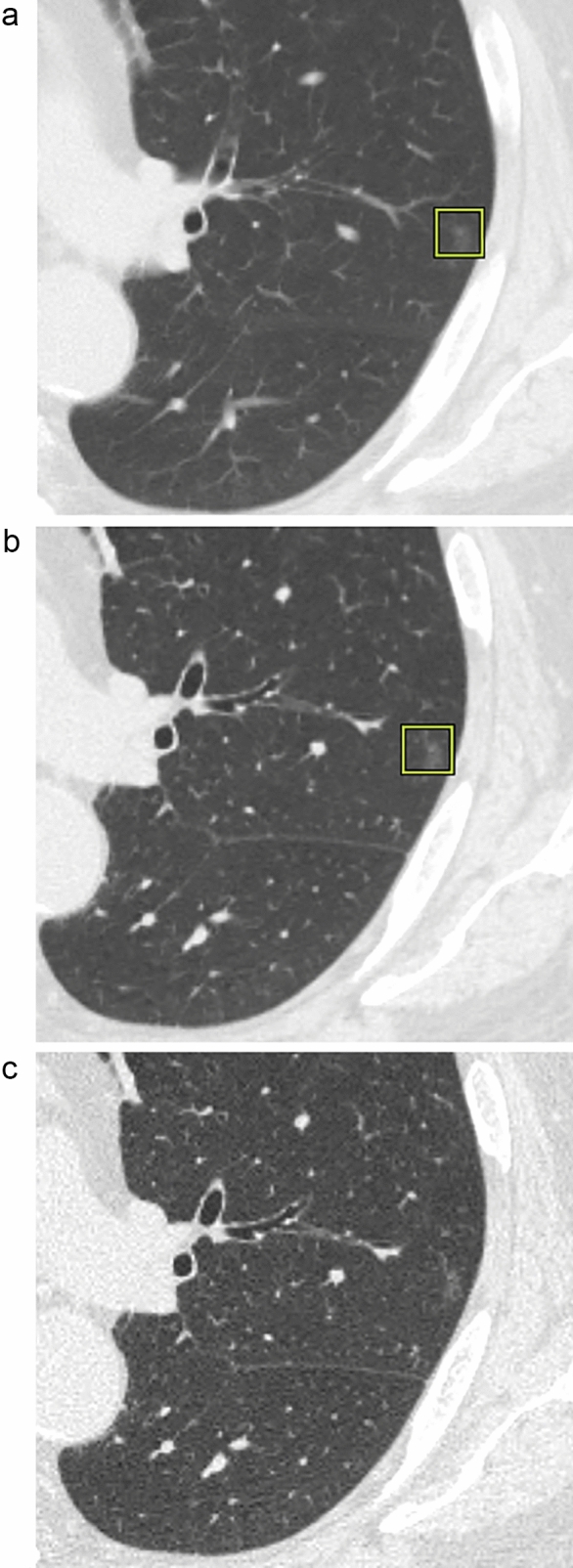
A 4-mm GGN in the left upper lobe was set as reference nodule (true nodule) by the radiologists. It was detectable by the DL-LND system on 5- and 1-mm slices (**a, b**), but it was not detectable on 0.25-mm because the GGN were obscured on thinner slices (**c**)

The sensitivity and the PPV on 5-mm slices were 79.8% (150/188) and 46.4% (150/323); they were 91.5% (172/188) and 34.8% (172/494) on 1-mm slices and 86.7% (163/188) and 36.1% (163/452) on 0.25-mm slices (Fig. 2). The sensitivity was greater on 1- and 0.25-mm slices than on slices with a thickness of 5 mm; on 0.25-mm slices it was not significantly better than on 5-mm slices (5-mm vs 1-mm;* p* < 0.01, 5-mm vs 0.25-mm; *p* = 0.07, 1-mm vs 0.25-mm; *p* = 0.14). Some sub-solid nodules were obscured on 0.25-mm slice images and they were not detected by the DL-LND system (Fig. 3). The PPV was significantly lower on 1- than 5-mm slices (5-mm vs 1-mm; *p* < 0.01, 5-mm vs 0.25-mm; *p* = 0.07, 1-mm vs 0.25-mm; *p* = 0.14)**.**

The sensitivity for small lung nodules (4–10 mm) on 5-mm slices were 72.7% (88/121); they were 89.3% (108/121) for 1-mm slices and 81.8% (99/121) for 0.25-mm slices (5-mm vs 1-mm; *p* < 0.01, 5-mm vs 0.25-mm; *p* = 0.09, 1-mm vs 0.25-mm; *p* = 0.10). The sensitivity for sub-solid nodules on 5-mm slices were 69.0% (69/100); they were 87.0% (87/100) for 1-mm slices and 80.0% (80/100) for 0.25-mm slices (5-mm vs 1-mm; *p* < 0.01, 5-mm vs 0.25-mm; *p* = 0.07, 1-mm vs 0.25-mm; *p* = 0.18). The sensitivity for small- and sub-solid nodules was the highest on 1-mm slices and 0.25-mm slices failed to improve its performance.

**Fig. 4:**
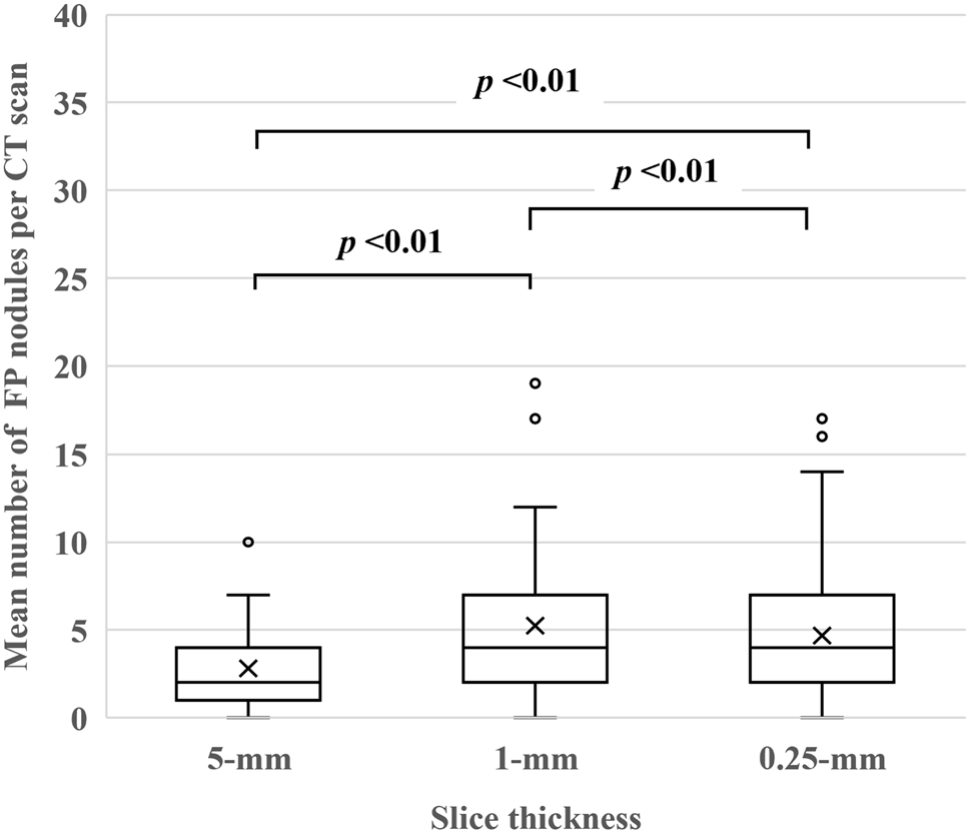
The mean number of FP nodules on 5-, 1-, and 0.25-mm slices were 2.8 (SD: 2.1), 5.2 (4.5), and 4.7 (3.8) per CT scan; the difference among the three slice thicknesses was significant (all: *p* < 0.01)

**Fig. 5:**
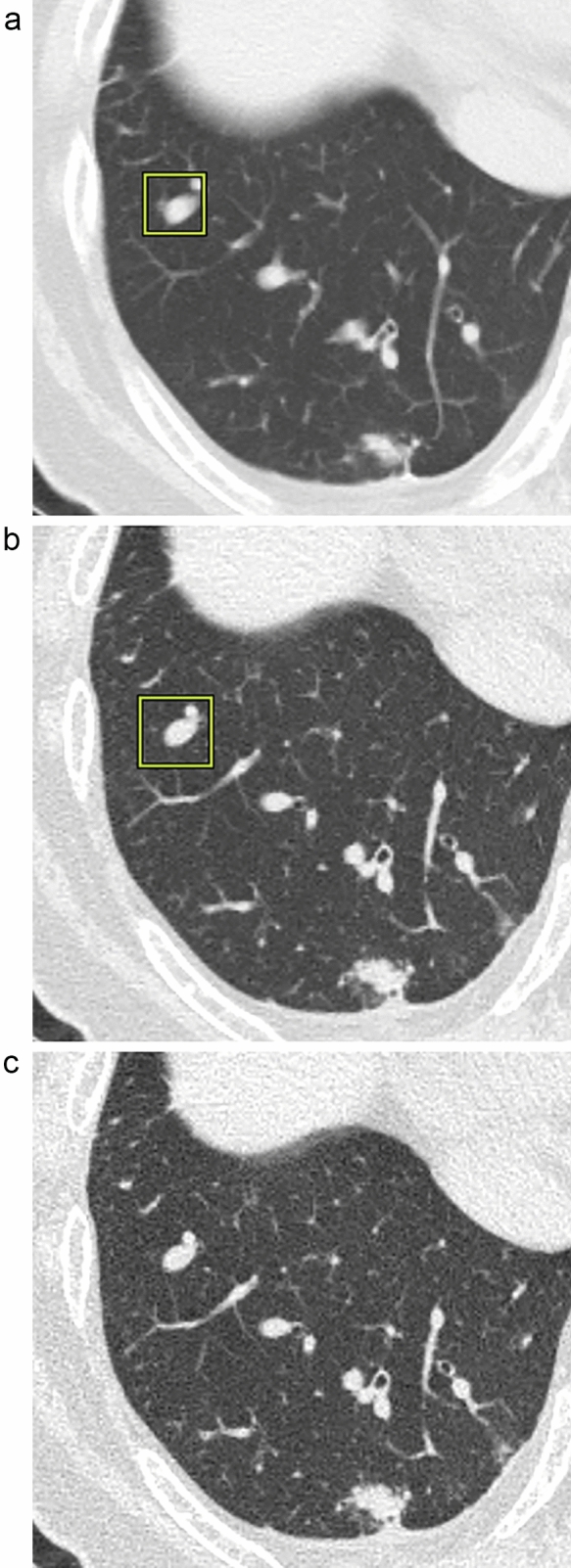
A nodule-like shadow was seen in the right lower lobe. It was pulmonary vasculature and classified as false nodule by the radiologists. On 5- and 1-mm slices (**a, b**), it was mis-identified as a lung nodule by the DL-LND system, but it was not detected on the 0.25-mm slices (**c**)

The number of FP nodules was 177 on 5-, 330 on 1-, and 295 on 0.25-mm slices. The mean number of FP nodules on 5-, 1-, and 0.25-mm slices was 2.8 (SD: 2.1), 5.2 (4.5), and 4.7 (3.8) per CT scan; the difference among the three slice thicknesses was significant (all: *p* < 0.01) (Fig. 4). Some false nodules detected by the DL-LND system on 1-mm slices were not detected on 0.25-mm slices (Fig. 5).

The processing time required by the DL-LND system to detect lung nodules was approximately 1 min per case; it was the same for all slice thicknesses.

## Discussion

We examined the performance of the DL-LND system using U-HRCT and three slice thicknesses ranging from 5 to 0.25 mm. The sensitivity on 1-mm slices was 91.5% and it was highest among the three slice thicknesses. The PPV on 1-mm slices was the lowest (34.8%) and the mean number of FP nodules per CT scan was the highest (5.1), nevertheless, the sensitivity on 1-mm slices were higher more than 10% than on 5-mm slices. The primary goal of AI algorithms for lung nodules detection is to increase the nodule-detection rate for the screening procedures [11]. In fact, the attached document for the DL-LND system used in this study states that its purpose is to reduce the number of lung nodules missed by radiologists. Therefore, the sensitivity is the most important factor in evaluating the performance of the DL-LND system, and we concluded that 1 mm was the best slice thickness for U-HRCT images. To examine the utility of thin slice thickness images, the sensitivity for small nodules (4–10 mm) and sub-solid nodules were also investigated. However, a slice thickness of 0.25 mm failed to improve its performance contrary to our hypothesis.

For lung cancer screening and for the management of incidental pulmonary nodules, the Fleischner Society [14] and the 1.2023 version of the National Comprehensive Cancer Network (NCCN) [15] recommend a slice thickness less than 1 mm due to the superior spatial resolution of such images and the minimization of partial volume effects; together, they improve the visibility and evaluation of small nodules. However, it was not known whether even thinner slices of 0.25 mm submitted to the DL-LND system would increase the detectability of lung nodules. Our findings indicate that a slice thickness of 0.25 mm failed to improve its performance.

There are several reasons that 0.25-mm slices failed to improve the performance of DL-LND system. First, we determined the cutoff size of the reference standards to 4 mm based on Lung-RADS and the earlier studies [1, 13]. Therefore, we considered the spatial resolution on 1-mm slices high enough for nodule detection. Second, the DL-LND system we used was trained on chest CT images acquired in 1997 and included an LIDC/IDRI dataset [16] but no 0.25-mm-slice images. As shown in Fig. 3, there were some sub-solid nodules which were obscured on 0.25-mm slice images and they were not detected by the DL-LND system. This may have contributed to the lower sensitivity for lung nodules on 0.25 mm images. Third, the image noise on 0.25-mm slice images was significantly increased due to the relatively insufficient incident photons on smaller detectors; this may have hampered the performance of the system. While slices as thin as 0.20 mm can be used for photon-counting detector CT (PCD-CT) studies [17], problems similar to those we encountered may arise.

The number of FP nodules was lower on 0.25-mm slices than on 1-mm slices. As shown in Fig. 5, the DL-LND system mis-identified the pulmonary vasculature in a right lower lobe as lung nodules on 5- and 1-mm slices, but not on 0.25-mm slices. This may be the potential advantage of 0.25-mm slice images that accurately recognize the nodule characteristics since the FP nodules are the one of the most significant issues for the DL-LND system and it increases the burden on radiologists [1]. The latest LD-LND systems not only detect lung nodules but they can also analyze their characteristics and differentiate malignant—from benign nodules [18–20]. As the nodule characteristics are presented accurately on 0.25-mm-slice images [7], it may be useful for AI algorithms to assess these characteristics of lung nodules.

The DL-LND system used in this study is one of the most widespread commercially available system which used more than 200 institutions. It was internally well-validated during the development phase and achieved sensitivity of 99.0% and 96.0% at 5.9 and 7.3 false positive nodules per scan on two public dataset [16]. However, the external validation using actual clinical data is insufficient. Fukumoto W. et al. externally validated that the sensitivity for lung nodules including lung cancer by this DL-LND system was very high with 96.0% using low-dose CT in LCS [21]. To the best of our knowledge, this is the first study which validated the performance of the DL-LND system using 0.25-mm thick U-HRCT images clinically. Although its performance needs to be improved, we confirmed that 0.25-mm slices were available for the DL-LND system with the same processing time as other slices.

This retrospective study has some limitations. First, the data we used were collected at a single institution and involved a small sample size. Therefore, further studies involving various institutions and larger datasets are required; however, U-HRCT is not yet widely available. Second, the reference standard was set using 1-mm slice images; this may affect the performance of the DL-LND system on other thick slices. However, the current standard slice thickness is 1 mm. Besides, there were no true positive nodules other than the reference standard nodules detected only on 5- and 0.25-mm slices. Third, we used 512 matrices reconstructions for 0.25-mm slices because the huge amount of data required for 1024 or 2048 matrix reconstructions puts excessive stress on the server and is not practical in the clinical setting. Lastly, as the recommended DL-LND parameters do not prescribe a slice thickness of 0.25 mm; appropriate training must be on such a slice thickness.

We concluded that, with the commercially available DL-LND system applied to U-HRCT scans, 1 mm was the optimal slice thickness for lung nodule detection.

## Abbreviations

AI: Artificial intelligence
U-HRCT: Ultra-high-resolution CT
DL-LND: Deep-learning-based lung nodule detection
PPV: Positive predictive value
FP: False positive
CAD: Computer-aided detection
LIDC/IDRI: Lung Image Database Consortium and Image Database Resource Initiative
PCD-CT: Photon-counting detector CT
FOV: Field of view
SD: Standard deviation
GGN: Ground-glass nodule
HU: Hounsfield unit
